# Skeletal Muscle in Cerebral Palsy: From Belly to Myofibril

**DOI:** 10.3389/fneur.2021.620852

**Published:** 2021-02-18

**Authors:** Jason J. Howard, Walter Herzog

**Affiliations:** ^1^Nemours-Alfred I. duPont Hospital for Children, Wilmington, DE, United States; ^2^Faculty of Kinesiology, University of Calgary, Calgary, AB, Canada

**Keywords:** cerebral palsy, skeletal muscle, muscle contractures, muscle morphology, spasticity, satellite cells, connective tissue, epigenetics

## Abstract

This review will provide a comprehensive, up-to-date review of the current knowledge regarding the pathophysiology of muscle contractures in cerebral palsy. Although much has been known about the clinical manifestations of both dynamic and static muscle contractures, until recently, little was known about the underlying mechanisms for the development of such contractures. In particular, recent basic science and imaging studies have reported an upregulation of collagen content associated with muscle stiffness. Paradoxically, contractile elements such as myofibrils have been found to be highly elastic, possibly an adaptation to a muscle that is under significant *in vivo* tension. Sarcomeres have also been reported to be excessively long, likely responsible for the poor force generating capacity and underlying weakness seen in children with cerebral palsy (CP). Overall muscle volume and length have been found to be decreased in CP, likely secondary to abnormalities in sarcomerogenesis. Recent animal and clinical work has suggested that the use of botulinum toxin for spasticity management has been shown to increase muscle atrophy and fibrofatty content in the CP muscle. Given that the CP muscle is short and small already, this calls into question the use of such agents for spasticity management given the functional and histological cost of such interventions. Recent theories involving muscle homeostasis, epigenetic mechanisms, and inflammatory mediators of regulation have added to our emerging understanding of this complicated area.

## Introduction

With respect to its musculoskeletal manifestations, cerebral palsy (CP) has been defined as a static encephalopathy, typified by muscle contractures (i.e., shortening) and bony deformities that are progressive with growth (1). The majority of children with CP exhibit a spastic motor type, whereby muscle stiffness increases proportional to the velocity of induced stretch (2, 3). The “spastic catch” that results, prior to the onset of fixed muscle shortening, has been referred to as a dynamic contracture, more commonly encountered in younger children with CP (4, 5). With time, the muscle develops a “static contracture” (i.e., the muscle becomes short) (6). As an overt manifestation of CP—associated with gait abnormalities, joint dislocations, seating issues, and impediments to activities of daily living (ADL)—muscle contractures (both dynamic and static) have been a convenient, and obvious, target for clinicians, both by nonsurgical and surgical means (7).

**Figure 1:**
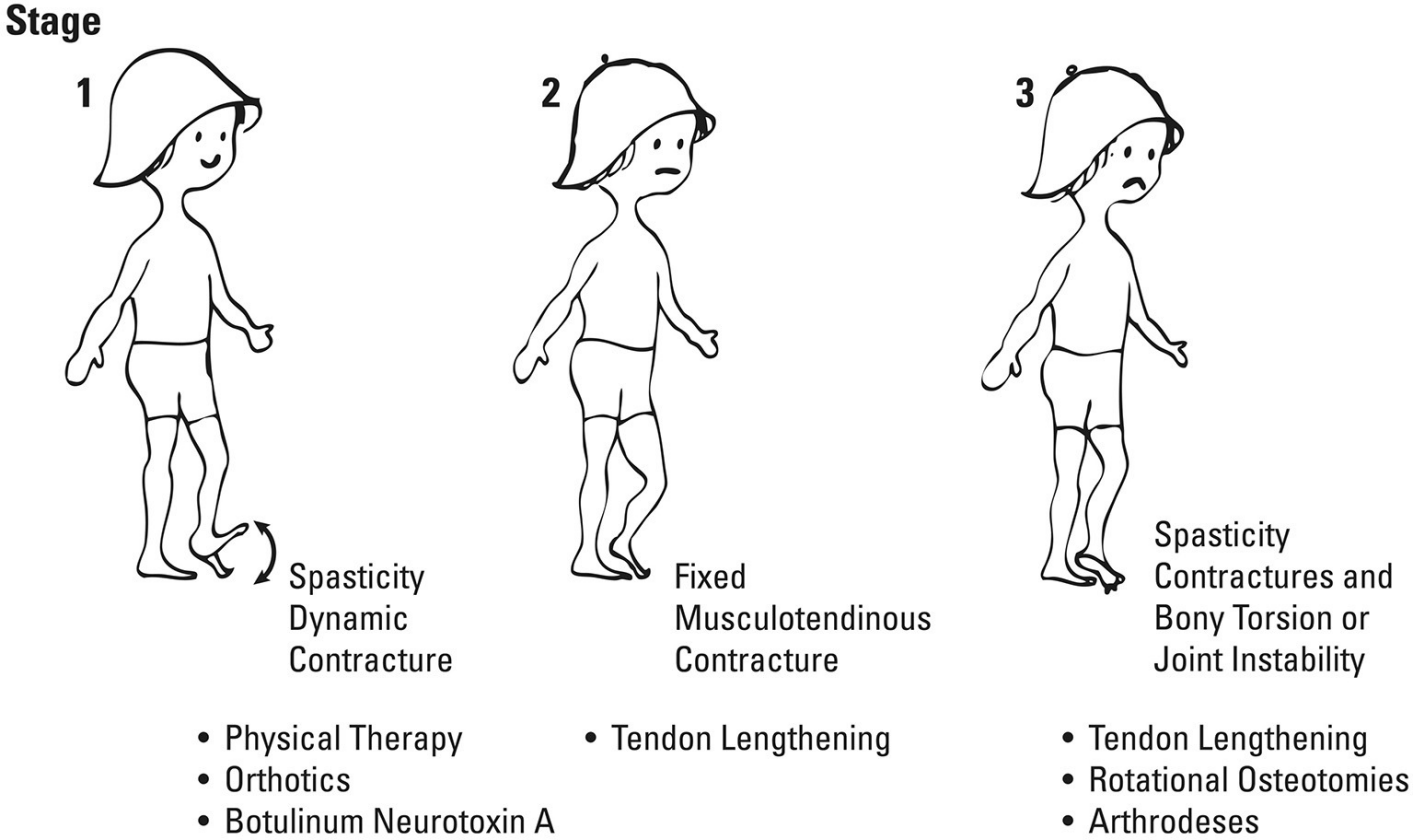
Rang's three phases of cerebral palsy, the so-called “Traditional View,” which emphasizes spasticity as a primary driver of both muscle and bony deformities. (Used with permission ^©^Bill Reid/Kerr Graham, RCH Melbourne).

Given the natural history of muscle contracture development, with dynamic contractures preceding static, along with the concomitant development of bony deformities, it was natural to conclude that spasticity was the driving force behind these manifestations. As such, treatments focused on reducing spasticity, most notably injection with botulinum toxin, have been instituted at an early stage with the goal of preventing static contractures and bony deformities and thus orthopedic surgery (8). This treatment approach, effectively communicated by Dr. Mercer Rang in his three stages of CP (9) (Figure 1), has been recently referred to as the “Traditional View” (10, 11), providing an intuitive means by which these children have been treated over the last 25 years.

The “Traditional View” presupposed that skeletal muscle in CP was merely an end organ in CP, its abnormal activation causing protracted postures eventually leading to fixed contracture, all secondary to hypertonia from the primary brain insult. However, recent developments in our understanding of the CP muscle challenge that view. More than just an end organ, intrinsic changes in the CP muscle pathomorphology—including abnormalities in sarcomerogenesis, decreased satellite cell number and function, increased connective tissue and fat, highly elastic myofibrils, and ribosomal dysfunction—suggest a more localized role in the development of contracture (12–15). Indeed, epigenetics has become an important area of interest, with abnormalities in DNA methylation being correlated to the diagnosis of CP in general and more specifically to disordered ribosomal-mediated protein synthesis, proportional to disease severity (16, 17).

Coupled to clinical research showing that the treatment of spasticity does not reliably prevent the development of static muscle contractures and bony deformities or the need for orthopedic surgery, it is very likely that the *Traditional View* is incorrect, with primary muscle deformities developing in tandem with spasticity rather than as a result of abnormal muscle activation (18–24). Indeed, impaired muscle growth mismatched to more normal increases in bone length, along with significant increases in intramuscular connective tissue, would seem to be more primary determinants of static contracture development in CP.

As such, the goal of this review is to summarize what is currently known about the pathophysiology of the CP muscle and how it relates to the musculoskeletal manifestations of the disease. In addition, the effects of various common treatments on the intrinsic properties of the CP muscle will be explored.

## The Cerebral Palsy Phenotype: Positive and Negative Aspects

**Figure 2:**
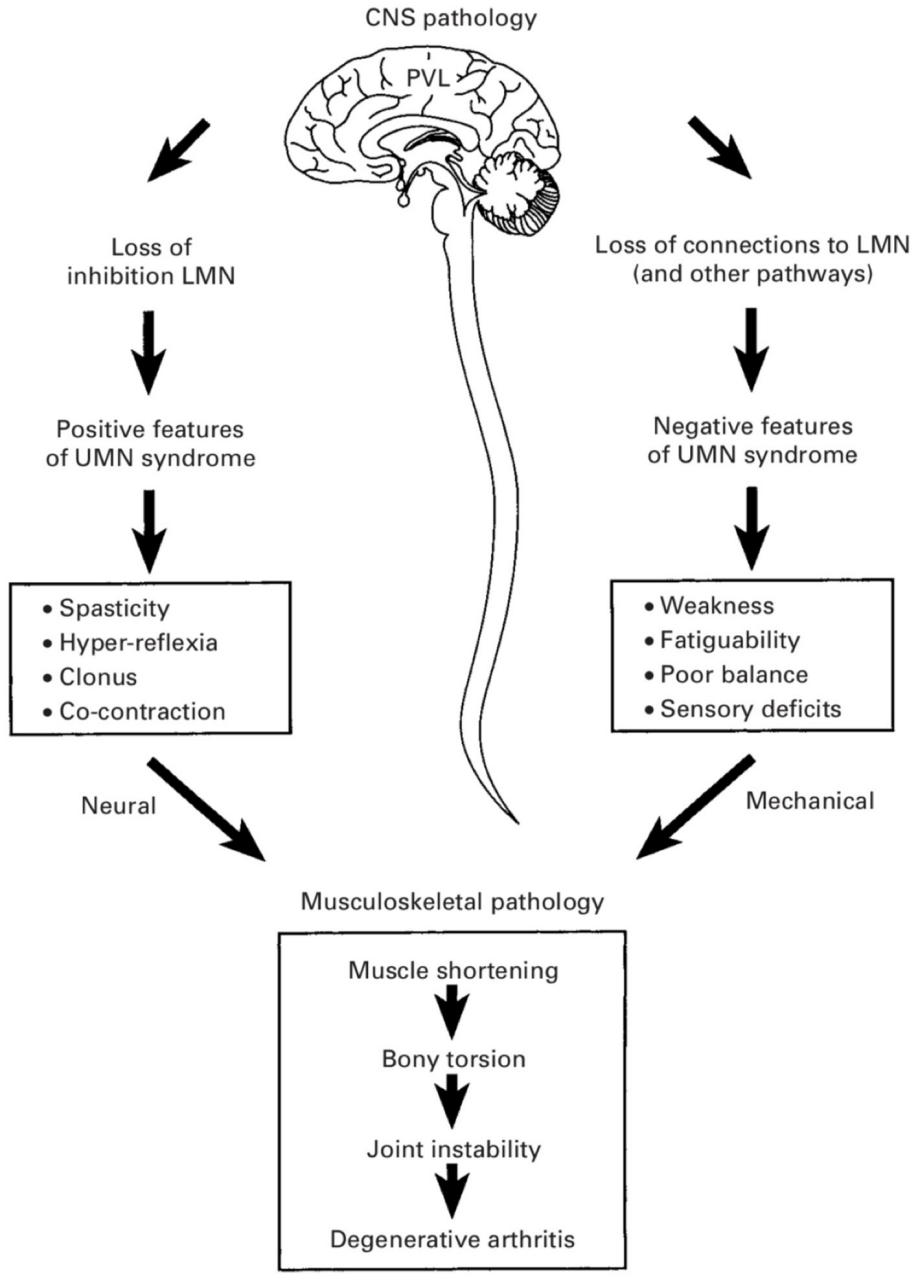
Positive and negative features of the upper motor neuron syndrome in cerebral palsy (CP). The negative features—including weakness, poor balance, and impaired selective motor control—are considered to be more important determinants of gross motor function in CP and are more likely causes of bony deformities and joint instability. (Used with permission © Bill Reid/Kerr Graham, RCH Melbourne).

Depending on the etiology of neurological insult, children with CP exhibit several different motor types, both hypertonic and hypotonic in nature. The most common motor type, and thus the most commonly investigated, is “spastic,” with 86% of a population-based CP cohort being of this type (2). As a velocity-dependent increase in dynamic muscle stiffness, the manifestations of spasticity are readily appreciable both on physical examination and by parents. It is natural then to attribute these manifestations to the motor type itself, described above as the “Traditional View” and hence divert focus and resources to rectifying spasticity. In addition to the “positive” features of the upper motor neuron syndrome that typifies the CP phenotype (including spasticity, hyperreflexia, clonus, and muscle cocontraction), the negative features (most notably weakness but also poor selective motor control and balance) have been implicated as the primary determinants of gross motor function (5) (Figure 2). As will be discussed in the sections below, properties intrinsic to the sarcomere in the CP muscle seem to be causative in this respect, being both overlong and subject to a high rate of excursion.

According to Rang's Traditional View, it has been widely accepted that spastic muscles exert abnormal forces on growing bone, causing bony deformities—in the case of the hip—that include coxa valga (increased femoral neck-shaft angle), increased internal femoral torsion (anteversion), and acetabular dysplasia (i.e., hip displacement) (25). A recent population-based study of children with CP disputes this view, revealing persistent increases in femoral anteversion (present from birth) and coxa valga, both associated with disease severity according to the Gross Motor Function Classification System (GMFCS) (26). Another study showed no difference in the incidence of hip displacement, regardless of whether the motor type was hypertonic or hypotonic (27). Thus, current thinking suggests that abductor insufficiency and a lack of weight bearing are responsible for primary deformities of the proximal femur leading to hip displacement rather than from the positive features of CP (28).

With respect to ambulatory patients with CP, eccentric, antigravity muscles—such as the soleus—are of primary importance, modulating the site of application of the ground reaction force on the foot during weight bearing and allowing for biomechanically advantageous ambulation. Crouch gait, whereby the ankle is in substantial dorsiflexion, the knee and hip are flexed, and the ground reaction force is behind the knee develop as a consequence of either natural or iatrogenic weakness of the soleus. Necessitating high knee extension moments, this gait pattern has a high energy cost and is largely unsustainable (29). As will be discussed in later sections, understanding the mechanisms by which force is decreased—or potentially enhanced—in antigravity, eccentric muscles like soleus is important to avoid treatment interventions that might further compound the problem of muscle weakness in CP (e.g., aggressive surgical lengthening or chemical denervation).

Before one can understand the CP muscle, however, one must first understand typically developing muscle.

## Skeletal Muscle Basics

Muscles are the engines of life. They allow us to move, to breathe, to play, and to function in everyday life. When muscles are properly trained and coordinated, they allow us to run 100 m in 9.58 s, play Liszt's “La Campanella,” or perform a reverse 4 1/2 somersaults dive in the pike position. Muscles are the organs of strength and power. Their intricate and highly specialized structure is aimed at producing mechanical work from metabolic energy in the most effective and most efficient manner.

### Muscle Morphology

**Figure 3:**
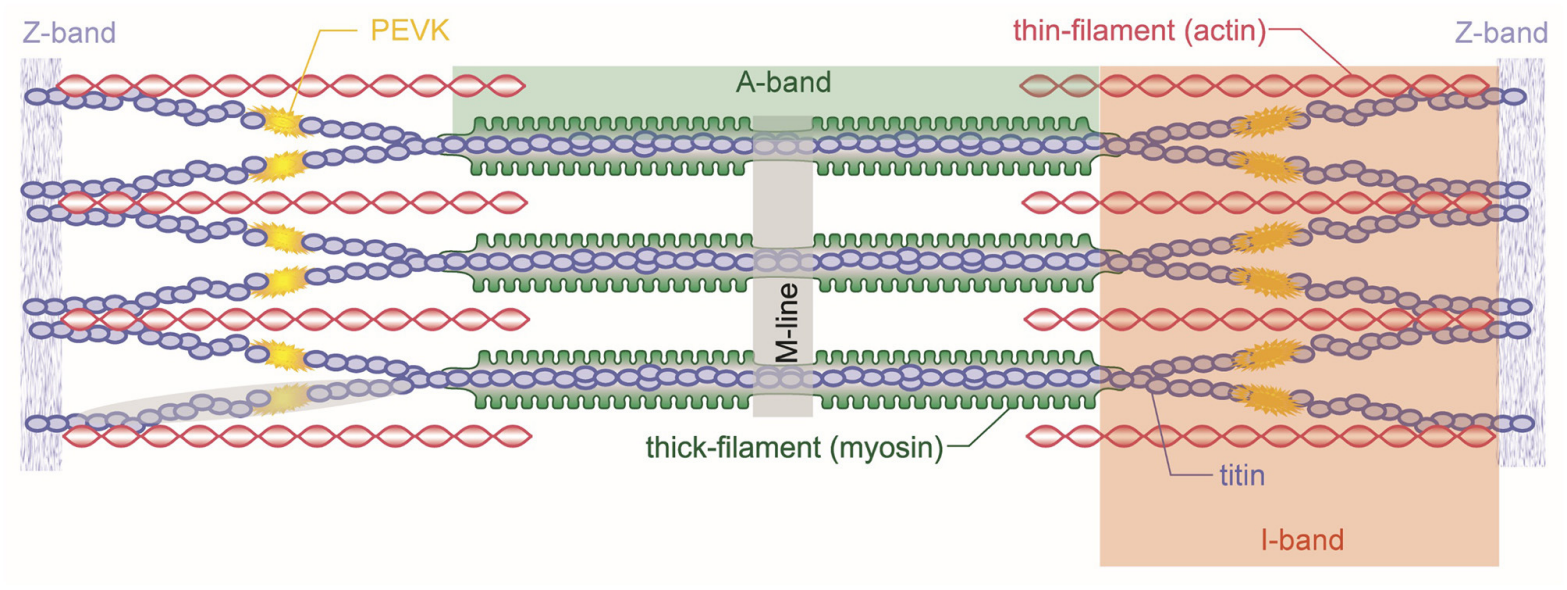
Schematic representation of a skeletal muscle sarcomere. Shown are the contractile filaments actin and myosin and the structural protein titin. Sarcomeres are bordered by the Z-bands from which titin and actin emanate. Myosin is centered in the middle of the sarcomere and held in place by the titin filaments.

The basic contractile unit of a skeletal muscle is the sarcomere (Figure 3). Sarcomeres are repeating structures of intricately aligned contractile and structural proteins, bounded by the so-called Z-band. Sarcomeres are made up of the contractile proteins actin and myosin and a series of structural proteins including titin, nebulin, desmin, and dystrophin. The myosin (or thick) filament is located at the center of the sarcomere, while the actin (or thin) filament merges at either end of the sarcomere into the Z-bands from where it reaches toward the center of the sarcomere. Depending on the length of the sarcomere, which in turn depends on the length of the muscle, the actin and myosin filaments overlap to different degrees, which is an important consideration in the amount of force a sarcomere (or the muscle) can exert.

**Figure 4:**
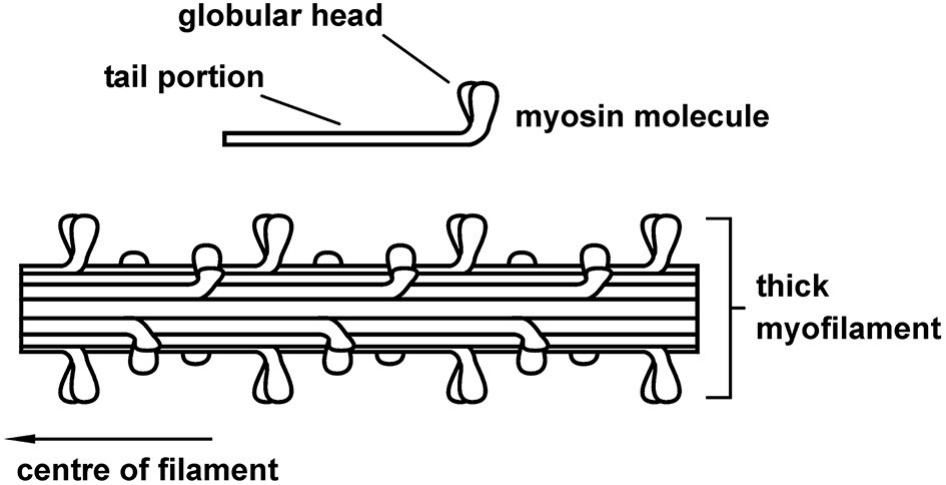
Schematic representation of the thick (myosin) skeletal muscle filament comprised primarily of myosin proteins. Myosin had a tail portion that is incorporated into the main body of the thick filament and two globular heads that reach out from the myosin toward the actin filament. Cross-bridges form cyclic attachments with actin, pulling actin past the myosin filament, thereby producing muscle contraction and force.

**Figure 5:**
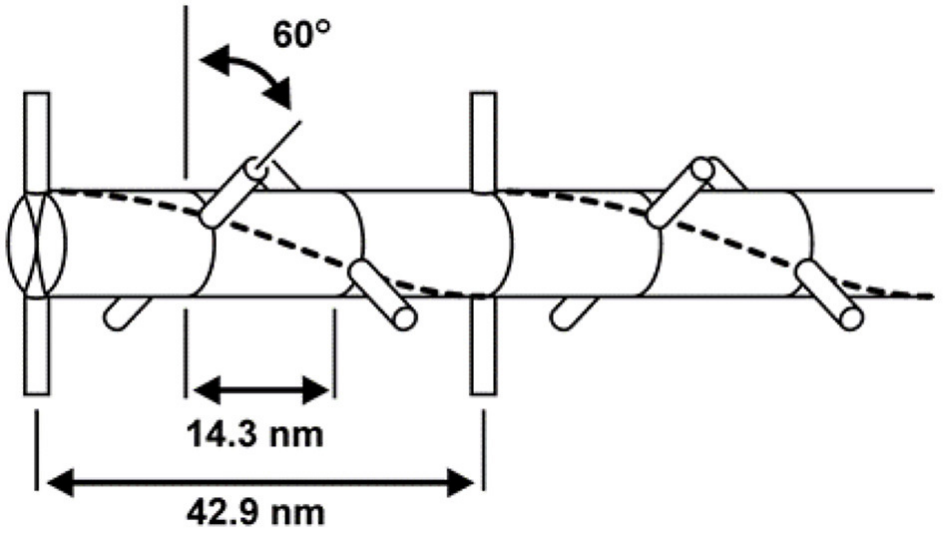
Geometric arrangement of the cross-bridges on the myosin filament. Cross-bridges come in pairs that are offset by 180° and are arranged every 14.3 nm along the myosin filament. Neighboring pairs of cross-bridges are rotated by 60°; therefore, cross-bridges with the same orientation (and thus attaching to the same actin filaments) occur every 42.9 nm.

The myosin filament, as its name implies, is primarily made up of myosin molecules (Figure 4). Myosin molecules have a tail portion that forms the structural part of the thick filament and a globular head part that emerges from the thick filament and is used to attach to specific sites on actin and pull the actin past the myosin filament upon muscle activation and contraction. These globular head portions of the myosin molecule are referred to as cross-bridges. Cross-bridges in mammalian skeletal muscles are arranged in a uniform manner, with actin filaments arranged around each myosin filament in a hexagonal array (Figure 5), each half myosin filament supplying six actin filaments with cross-bridges, 60° apart from each other.

**Figure 6:**
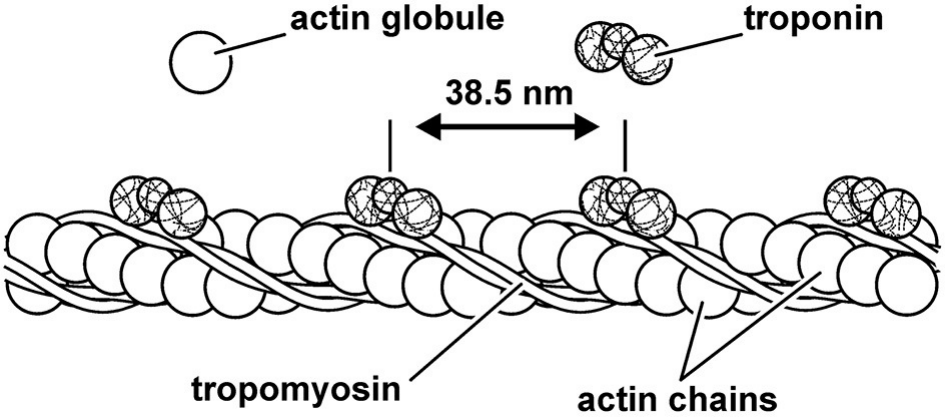
Schematic representation of the thin (actin) filament. Actin is made up of two serially linked chains of actin globules and the repeating structures of the regulatory proteins tropomyosin and troponin. The regulatory protein troponin C binds calcium upon muscle activation, resulting in a configurational change of the troponin–tropomyosin complex that allows for cross-bridge attachment to actin.

The actin filament is made up of two chains of serially linked actin globules (Figure 6). Actin also contains important regulatory proteins, so named because of their role in controlling myosin cross-bridge attachment to actin. The first of these regulatory proteins is the troponin tricomplex, which repeats every 36–39 nm on the actin filament. The troponin tricomplex consists of troponin C, troponin T, and troponin I proteins. Upon muscle activation, calcium is bound to troponin C, which in turn causes a conformational change in the troponin/tropomyosin complex, exposing the actin attachment site to the myosin cross-bridges, thus allowing contraction and force production.

**Figure 7:**
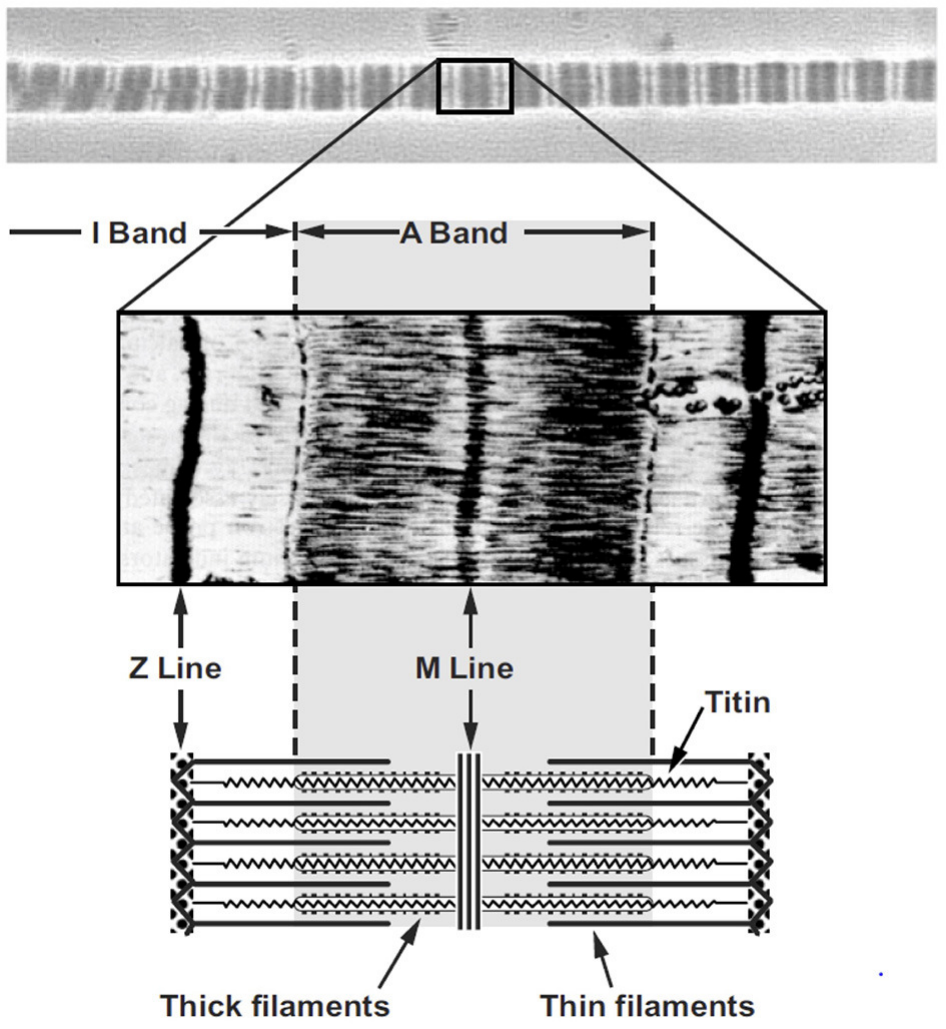
Micrograph of a single myofibril (top panel) with the typical dark (A-band) and light (I-band) striation pattern and a corresponding sarcomere (middle panel) with a schematic representation of the sarcomere (bottom panel) including the appropriate labeling.

Myofibrils are the contractile organelles in a muscle fiber, being made up of serially arranged sarcomeres (Figure 7). For typically developing human muscle, sarcomeres exert force most optimally at a length of ~2.6 μm (30, 31). Therefore, each 1 mm (=1,000 μm) of myofibril contains about 400 sarcomeres perfectly aligned in series with each other. Since myofibrils can easily be several centimeters in length, a myofibril can contain thousands of sarcomeres. Myofibrils are between 0.5 and 1.0 μm in diameter and thus are ~1/100th of the diameter of a human hair.

**Figure 8:**
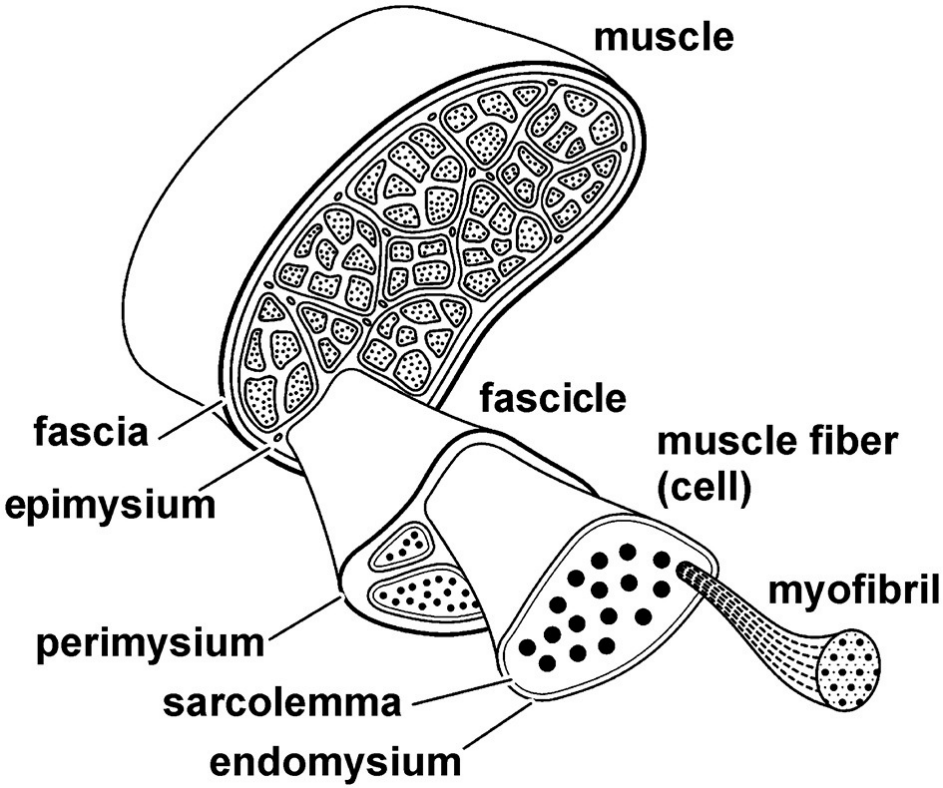
Schematic structural hierarchy of a skeletal muscle. The entire muscle is made up of fascicles, fascicles are made up of fibers (cells), and fibers are comprised of myofibrils. Each structural entity of the muscle is surrounded by connective tissues that connect the various structural levels with each other and connect the entire muscle to other muscles, bones, and fascia. The connective tissue layers are referred to as the epimysium, perimysium, and endomysium, as indicated in the figure.

Muscle fibers are long and thin multinucleated cells of skeletal muscles (Figure 8). They typically contain several thousand myofibrils that lie in parallel to each other. Muscle fibers are surrounded by a cell membrane, the sarcolemma, and connective tissue, the endomysium that connects neighboring muscles fibers. The next greater structural entity is the muscle fascicle (Figure 8). Muscle fascicles are made of multiple muscle fibers, and they are contained within a connective tissue layer called perimysium. Finally, the whole muscle is made up of muscle fascicles. Muscles are surrounded by connective tissues that connect one muscle of a group to the other muscles in that same group. The connective tissue layer closest to the muscle is referred to as the epimysium, and other layers of connective tissue are referred to generically as muscle fasciae.

**Figure 9:**
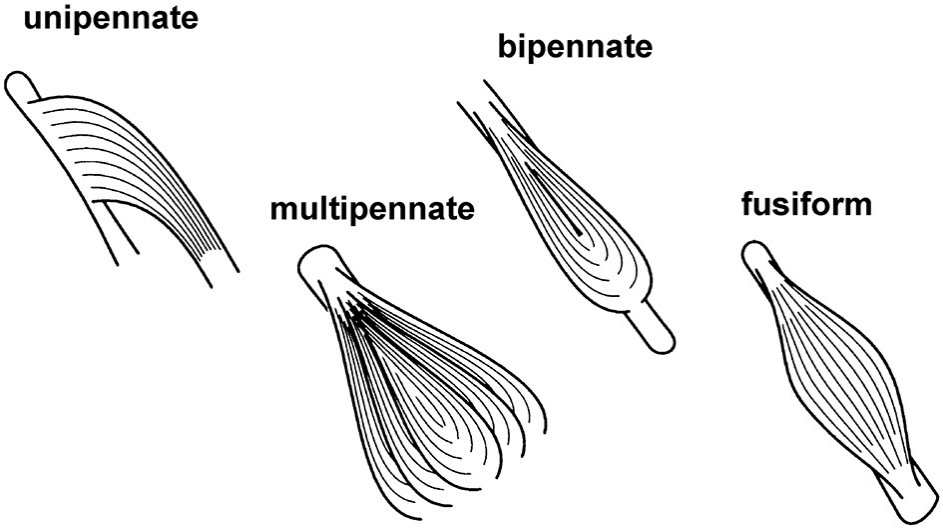
Schematic figure of different structural arrangements of fibers relative to the long axis of the muscle. See text for further explanation and definition.

Skeletal muscles have distinct arrangements of their fibers relative to the long axis of the muscle (Figure 9). Muscles whose fibers run essentially parallel to the long axis of the muscle are either referred to as fusiform or parallel fibered. When all fibers of a muscle are essentially aligned in the same direction and at a distinct angle to the long axis of the muscle, then the muscle is said to be unipennate. When there are two or more distinct fiber directions, a muscle is referred to as bipennate or multipennate. Examples of these different structural arrangements can be found in all animals, including humans: sartorius (parallel fibred), medial gastrocnemius (unipennate), rectus femoris (bipennate), and deltoid (multipennate). Typically, pennate muscles have shorter fascicles/fibers relative to the entire muscle length than parallel/fusiform muscles; thus, their fibers take up less volume and allow for more parallel fibers per volume than the fibers in parallel muscles. Functionally, this means that for two muscles of equal volume, a pennate muscle has a greater maximal force capacity (maximal force depends on the physiological cross-sectional area of the muscle) but a smaller excursion range (excursion depends on the length of fibers) over which it can produce force than a parallel fibered muscle, while the work potential (area under the maximal force-length curve) for the two muscles of equal volume is the same.

Muscles in the human body, and in most mammals, are of mixed fiber type. On the most basic levels, muscle fibers are grouped into slow twitch (type 1) or fast twitch (type 2) fibers. Fast twitch fibers are typically further subdivided into type 2a, 2b, and 2x based on their myosin heavy chain gene expression. However, there are hybrid expressions of myosin heavy chains that make for a virtually continuous spectrum of fast twitch fibers, but these will not be discussed further. Metabolically, the slow twitch type 1 fibers and the fast twitch type 2a fibers rely primarily on oxidative metabolism for force and work production, while the 2b and 2x fast fibers rely primarily on glycolytic metabolism. Functionally, as the name implies, slow twitch fibers have slow twitch characteristics, slow force rise and relaxation, and a slow speed of unloaded (maximal) shortening relative to the fast twitch fibers. Therefore, even though the maximal isometric force per cross-sectional area of slow and fast twitch fibers is similar, force during shortening is much greater in fast compared to slow twitch fibers, resulting in a maximal power output that is three to four times greater in fast compared to slow twitch fibers. The low power output of the slow compared to fast twitch fibers is offset by their fatigue resistance allowing slow twitch fibers to perform submaximal work for a much longer period of time than the fast fatiguing fast twitch fibers.

### Mechanisms of Contraction

Prior to the 1950s, muscle contraction was thought to occur through the shortening of the A-band region containing the myosin filaments in sarcomeres. However, in 1954, Andrew Huxley and Hugh Huxley (not related) arrived independently at the conclusion that muscle shortening and lengthening was not caused by myosin shortening but by the relative sliding of the actin to the myosin filaments. This discovery has been named the “sliding filament” theory (32, 33). Three years later, Andrew Huxley proposed the first mathematical model of how the relative sliding of these two myofilaments may occur; the cross-bridge theory (34). Despite many refinements and improvements of the cross-bridge theory in the past half century, much of the initial model has been retained to this day and is still used in many applications of muscle modeling and muscle function. In simple words, the cross-bridge theory might be summarized as follows: The myosin filaments have protrusions (cross-bridges) that attach cyclically to specific attachment sites on the actin filaments, and when attached, they pull the actin past the myosin filaments, thereby producing shortening and force. The energy for these processes is provided by adenosine-triphosphate (ATP) that is hydrolyzed into adenosine-diphosphate (ADP) and a free phosphate: one ATP per cross bridge cycle.

Muscle contractions are categorized into three conceptual groups, depending on the length change of the muscle during contraction. When a muscle is contracting and its length remains the same, the contraction is called isometric. When a muscle is shortening during contraction, thereby overcoming the external resistance and producing positive mechanical work, the contraction is called concentric, and when a muscle is actively producing force, but the external forces are greater than those produced by the muscle, and thus the muscle is elongated while activated, the contraction is referred to as an eccentric contraction. These contractile conditions are insofar important as they determine the forces that are (maximally) available to a muscle. For example, a muscle that is shortening (concentric contraction) has a reduced capacity to produce force compared to a muscle that is being stretched at the same rate (eccentric contraction) (35).

### Mechanical Properties of Skeletal Muscle

In most textbooks of muscle mechanics and physiology, authors refer to two basic mechanical properties: the force–length and the force–velocity relationship. However, there is a distinct third property of skeletal muscle: the history-dependent property, which manifests itself as the residual force enhancement and residual force depression property.

**Figure 10:**
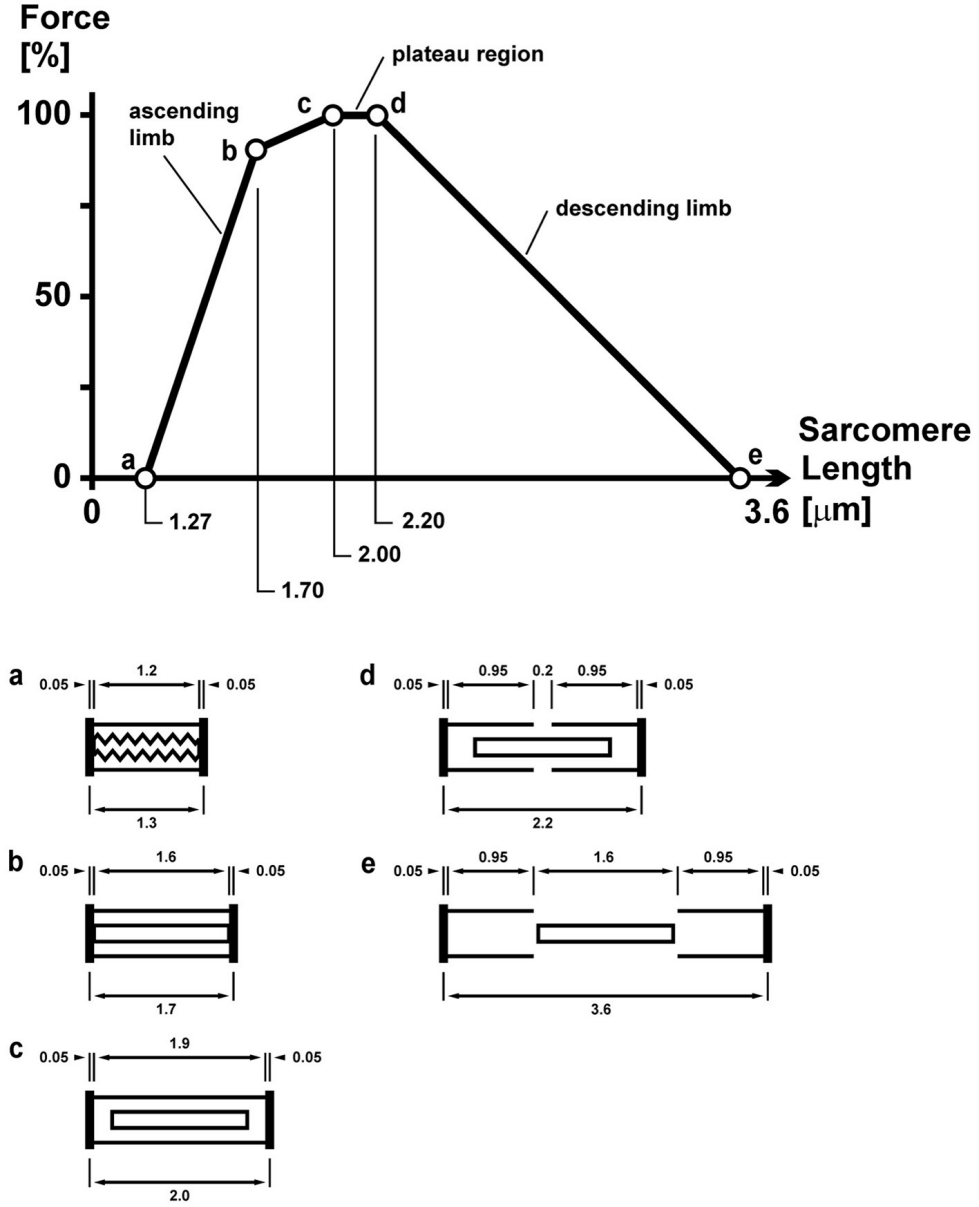
Sarcomere force–length relationship of a frog skeletal muscle as first described by Gordon et al. (38). The so-called plateau and descending limb regions are well explained by the sarcomere lengths (shown schematically below for lengths c, d, and e) and the corresponding overlap between the contractile filaments actin and myosin. The overlap between these filaments determines the number of possible cross-bridge interactions and thus the maximal, isometric force capacity.

The force–length relationship of skeletal muscles describes the maximal, active, steady-state, isometric force that a muscle can exert as a function of its length (36). Over a century ago, scientists were aware that the maximal force capacity of a muscle depended on its length (37). However, an understanding of the mechanisms underlying the force–length relationship only became available after the introduction of the sliding filament theory, when scientists proposed that the force potential of a muscle needs to be proportional to the amount of overlap that exists between the actin and myosin filaments in a sarcomere, and the amount of this overlap, of course, depends on the length of a muscle (38) (Figure 10).

**Figure 11:**
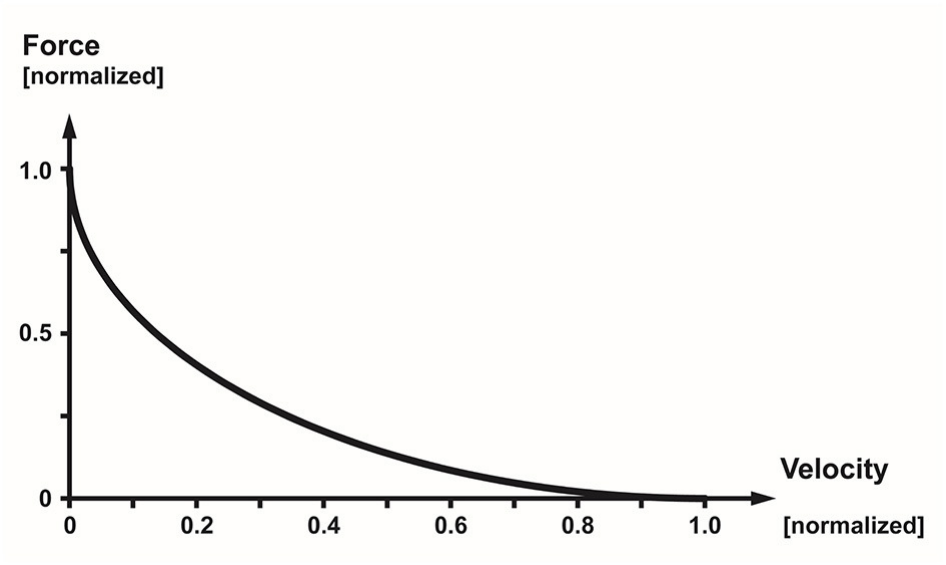
Normalized force–velocity relationship of a skeletal muscle showing the well-known hyperbolic relationship between the maximal, steady-state force of a muscle as a function of its speed of shortening.

The force–velocity relationship describes the maximal, active, steady-state force of a muscle at optimal length (the length where a muscle produces the maximal isometric force) as a function of its rate of change in length (typically referred to as the speed/velocity of shortening) (36) (Figure 11). Thus, it describes how the maximal steady-state force of a muscle decreases with increasing speeds of shortening. Fast twitch fibers can shorten faster than slow twitch fibers, and muscles with long fibers have a greater capacity to shorten fast than muscles with short fibers. The eccentric part of the force–velocity relationship is much less well defined than the concentric part, and is left out of this discussion here, but can be found in many textbooks of muscle mechanics and physiology (39).

**Figure 12:**
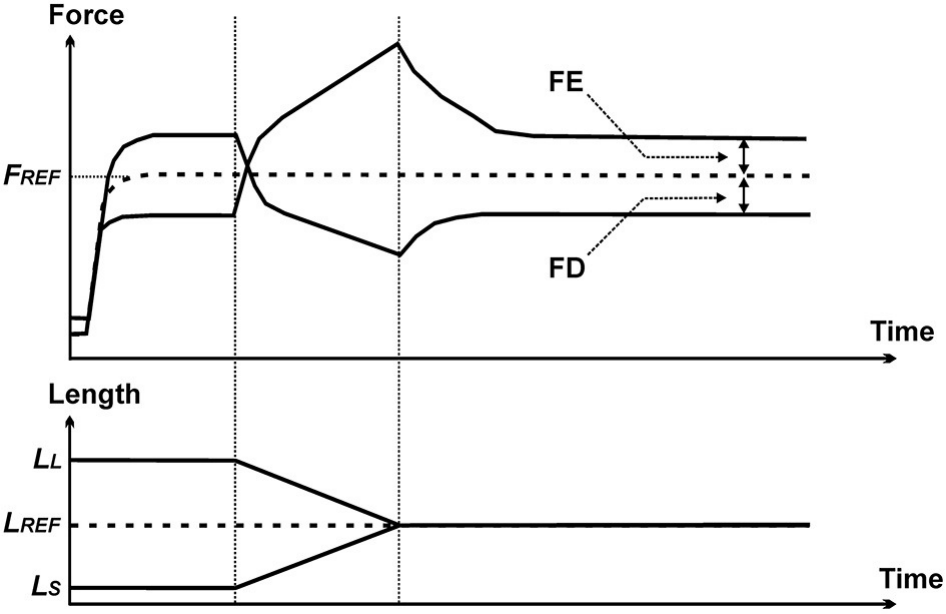
Force–time (top panel) and length–time (bottom panel) history of the contractile properties that produce residual force enhancement (FE) and residual force depression (FD). The dashed line labeled F_REF_ represents the force obtained in an isometric reference contraction. Force enhancement represents the increase in steady-state isometric force (relative to F_REF_) of a muscle following active stretching, and force depression is the decrease in steady-state isometric force (relative to F_REF_) following active shortening.

Residual force enhancement (rFE) and residual force depression (rFD) are two properties of skeletal muscle contraction that have been observed consistently for all skeletal muscles for more than half a century (40). rFE is the extra steady-state isometric force that is obtained when a muscle is actively stretched, compared to the corresponding (same length and same activation) purely isometric force (41) (Figure 12). rFD is the loss in steady-state isometric force observed following shortening of an active muscle compared to the corresponding (same length and same activation) purely isometric reference contraction (42) (Figure 12).

Residual force enhancement increases with increasing stretch magnitudes, is independent of the stretch speed, is not associated with a change in muscle stiffness (compared to isometric reference contractions), and can be abolished instantaneously by deactivating the muscle. It has been demonstrated that rFE is associated with the engagement of a passive structural element in skeletal muscle (43), and there is increasing evidence that this passive element is the structural, filamentous sarcomere protein titin (44–47).

Titin is a spring-like molecule that tethers the thick myosin filaments to the Z-line and is the primary determinant of myofibril stiffness (Figure 3) (48). The working hypothesis for titin's role in rFE is (i) that titin binds calcium upon activation, thereby increasing its spring stiffness by reinforcing molecular bonds that unfold when sarcomeres, and thus, titin are stretched, and (ii) that when a muscle undergoes an eccentric action (i.e., it is actively stretched), titin binds to actin so that its proximal segment cannot be elongated and its free spring length (the unbound distal segment) becomes shorter and thus stiffer (48–50).

## How Is Cerebral Palsy Muscle Mechanically Different?

CP muscle differs structurally, biologically, and mechanically from those of age-matched typically developing children (TDC). The primary function of skeletal muscles is to produce force and movement, and there is good evidence that spastic muscles in children with CP are reduced in these functions compared to TDC. There are at least four mechanical/structural reasons for the reduced active force potential of spastic skeletal muscles in children with CP:

- reduced muscle size,
- reduced contractile tissue,
- over-stretched sarcomeres, and
- loss of sarcomeric titin.

### Reduced Muscle Size

Muscle size is typically decreased in spastic muscles of children with CP compared to muscles in appropriate controls. Noble et al. (12) found an average muscle volume deficit of 27.9% in nine lower limb muscles of 19 independently ambulant children with spastic CP compared to typically developing children. Matthiasdottir et al. (51) found an average of 43% reduced fascicle length in 11 children with CP (ambulant and nonambulant) compared to 14 typically developing children, 6 of which were sex-matched twin siblings. However, physiological cross-sectional area, rather than muscle volume, is a direct indicator of maximal force capacity. Since physiological cross-sectional area can be estimated as volume divided by optimal fascicle length, and since fascicle lengths also tend to be reduced in children with CP, physiological cross-sectional area might not be as compromised as the reduced muscle volumes might suggest.

### Reduced Contractile Tissue

There are two basic mechanisms by which the amount of contractile tissue per unit of muscle cross-sectional area can be reduced: (i) through fibrosis (i.e., increased intramuscular connective tissue) (52) and (ii) by replacement of connective tissue by other tissues, primarily fat in the context of cerebral palsy (53).

Several authors have reported increased connective tissue in CP. Booth et al. (14) reported a significant increase in the hydroxyproline content (a proxy measure of collagen content) of vastus lateralis in children with spastic CP compared to TDC. deBruin et al. (54) reported increased connective tissue content in the perimysium with the flexor carpi ulnaris of children with CP (54). Lieber and Fridén (55) also reported increases in connective tissue in the CP muscle, but this was not consistent across all muscle groups. Furthermore, and somewhat surprising, the authors found that connective tissue content was only related weakly to passive forces in the muscles when compared at corresponding sarcomere length.

Replacement of contractile tissue in the CP muscle by fat has been shown to be prevalent, both in higher functioning and more severely affected children (53, 56). Noble et al. (57) studied 10 young adults with CP and GMFCS levels 1–3 and compared intramuscular and intermuscular fat content to a control group of typically developing young adults. They found a significant increase in intra- and intermuscular fat content in a variety of muscles in the patients with CP compared to control, even in the absence of recent treatments with botulinum toxin (BoNT-A), casting or surgical interventions. However, consistent longitudinal data on muscular fat invasion in patients with CP are not available. Interestingly, BoNT-A injection has been shown to significantly increase the amount of intramuscular fat, replacing atrophied contractile tissue, which may potentially compound the already compromised natural state of the CP muscle when used for the treatment of spasticity (58). This will be discussed in more detail below.

### Overstretched Sarcomeres

Human myofilament lengths are 1.65 μm for myosin and 1.27 μm for actin (31), resulting in an optimal sarcomere length for maximal force capacity between 2.64 and 2.81 μm (30, 59, 60). When sarcomere length in muscles are either shorter than 2.64 μm or longer than 2.81 μm, force capacity is reduced rapidly. Lieber et al. performed a series of studies measuring the *in vivo* sarcomere length in patients with spastic cerebral palsy and found consistent sarcomere lengths within the functional range of 3.5–4.0 μm, reducing the force capacity of these muscles to about 17–48% of maximum (30, 31). Similarly, we found average sarcomere lengths of 3.6 μm in the adductor longus of children with spastic CP compared to 2.6 μm in typically developing children (15). This increase in sarcomere length would be associated with a loss of ~55% in isometric force in the spastic adductor longus compared to control.

Interestingly, overstretched sarcomeres in children with cerebral palsy are often associated with reports of decreased fiber/fascicle lengths, thereby indicating a reduction in the number of serial sarcomeres in the fibers and myofibrils of children with cerebral palsy. A reduction in sarcomere number, in turn, is associated with a smaller excursion of the muscle and a greater absolute sarcomeric shortening speed for a given speed of muscle contraction. Both these factors would contribute to a further reduction in force generating capacity of the CP muscle (61). From a purely mechanical point of view, a reduction in serial sarcomeres, with the associated increase in sarcomere length and faster shortening speed, is likely the greatest contributor to force loss (i.e., weakness) in children with CP. Therefore, targeting interventions that might prevent the loss of serial sarcomeres in these patients would likely have the greatest effect on retaining/re-establishing the functional capacity of children with cerebral palsy.

### Loss of Sarcomeric Titin

The sarcomeric protein titin has been associated with many crucial functions in skeletal muscle sarcomeres: passive force (62), myosin and sarcomere stabilization (63), residual force enhancement (43, 64), passive force enhancement (46, 65), reduced energetic cost for muscle contraction (35, 66), and force transmission (67). There has been speculation that titin isoforms might be altered in children with CP, thus possibly contributing to the increased passive forces seen in biopsy samples from spastic muscles compared to healthy control muscles. However, these speculations have not been supported by recent evidence (15, 55). Paradoxically, in contrast to the increased passive forces observed in whole muscles, fascicles, and fibers (55), we found that passive forces in single, isolated myofibril preparations were reduced by 50% compared to control in adductor longus from children with CP undergoing hip adductor release surgery (15). This decrease in passive force was not associated, as we had initially speculated, with a change in the titin isoform, but was associated with a 50% decrease in the amount of titin in sarcomeres of the spastic muscle. Instead of the normal six titin molecules per half myosin filament (6:1 ratio), we found that, in these biopsies, the ratio of titin to half myosin was in the vicinity of 3:1. It has been shown previously that a decrease in the amount of titin per myosin is directly, and proportionally, associated with a corresponding decrease in active force (68). Therefore, we assume that the loss of titin in the spastic muscle of children with cerebral palsy is also associated with a loss of active force production, but this requires further investigation.

## Satellite Cells and Epigenetic Abnormalities

### Satellite Cells Are Reduced

In addition to the mechanical/structural aspects detailed above, disordered muscle growth and regeneration may be causative factors associated with the decreased muscle volumes seen in children with CP. Normal growth and healthy regeneration of muscle requires satellite cells, progenitor cells responsible for the production of myocytes, and thus contractile muscle tissue (69). Even though muscle size and fascicle lengths are often found to be smaller in CP compared to the muscle from TDC, from a functional point of view, the reduced number of serial sarcomeres in fibers and myofibrils is likely of primary importance.

Unfortunately, serial sarcomere adaptation and longitudinal muscle growth are much less studied and understood than parallel sarcomere adaptation and circumferential muscle growth. It is well accepted, however, that muscle stretching (70), increased muscle excursion (71), and long-term muscle length changes (72–75) are potent stimulators for sarcomerogenesis (i.e., serial sarcomere adaptation) in healthy skeletal muscle. However, even aggressive muscle stretching through physiotherapy and joint fixation has not been shown to promote normal longitudinal growth and sarcomerogenesis in the CP muscle, suggesting an intrinsic reason for the lack of response to mechanical stimuli.

One such reason may be related to satellite cell function. Lieber et al. found that CP muscles have a reduced number of satellite cells and, given their known role in myofiber development, suggested that this reduction might be causative for decreased longitudinal muscle growth (13, 76, 77). However, there is no direct evidence that the reduced number of satellite cells is the cause of the inhibited sarcomerogenesis or merely a by-product. Similarly, it is not known if the reduction in satellite cells is specific to the CP muscle or if muscles not directly affected by the brain lesion (i.e., non-spastic) also have a reduced number of satellite cells (personal communication with R. Lieber, August 2019). If satellite cells were also reduced in nonspastic muscles, then likely they would not be the reason for impaired growth of spastic muscles. If, however, nonspastic muscles in children with cerebral palsy had a normal satellite cell concentration, then it would be strong evidence for a direct and local involvement of the satellite cells in the inhibition of muscle growth. This knowledge could then be used as a primary target strategy for local intervention with the aim to promote normal sarcomerogenesis in spastic muscles of children with cerebral palsy.

### Epigenetics: The Role of DNA Methylation

DNA methylation, whereby gene expression is modulated through the addition or subtraction of methyl groups, has become an area of increasing interest in CP, particularly with respect to impaired muscle growth and sarcomerogenesis (78). It has been hypothesized that epigenetic mechanisms, most notably DNA methylation, play a significant role in regulating the response of skeletal muscle in CP, determining the initiation, and/or severity, of contracture (79). Tissues with high metabolic demand and need for increased synthesis, such as skeletal muscle, typically have hypomethylated DNA translating to increased DNA transcription and vice versa (17, 80). These epigenetic changes have been further hypothesized to be imprinted at the time of the inciting insult in CP (11, 16). The literature supporting this theory is emerging but still indeterminate.

In an interesting study from Sweden, CP muscle samples from the biceps brachii determined that DNA methylation of the ribosomal DNA (rDNA) promotor responsible for ribosome biosynthesis—a function crucially important for muscle growth—was increased in more severely affected children (by GMFCS) and with more severe contractures (17). The study, however, did not find a difference in rDNA promotor methylation between CP and TDC subjects.

In another comparative study investigating the role of DNA methylation in the erector spinae muscles of CP and TDC children undergoing scoliosis surgery, significant differences in methylation patterns were found, suggesting an influence of this mechanism in skeletal muscle pathology in CP (81). Using machine learning techniques, this same research group demonstrated significantly different DNA methylation patterns in peripheral blood, reliably detecting CP from non-CP (16).

Investigating the impact of epigenetic changes on satellite cell-derived myoblasts in CP vs. TDC hamstring muscles, Domenighetti et al. (76) showed that, in addition to having a decreased capacity to form and fuse myotubes, the signaling pathway responsible for regulating myotube formation was correspondingly downregulated in children with CP. Accordingly, the promotor region of this pathway (integrin-β 1D) was found to be hypermethylated, with improved myotube formation following application of a demethylating agent. The authors proposed that DNA hypermethylation of myoblast gene promotor regions were responsible for the decreased myofiber formation (and thus muscle growth) observed in the CP muscle. They also suggested that this “epigenetic memory” has likely been imprinted very early in development, preceding the development of muscle contracture.

Although in the early stages of investigation, the role of epigenetics in CP muscle development seems to have merit, this may provide an explanation, or at least part of, as to why growth is compromised in this population.

## Pathophysiology of Spastic Muscle Contracture Treatment

### Botulinum Toxin and Skeletal Muscle Adaptations

**Figure 13:**
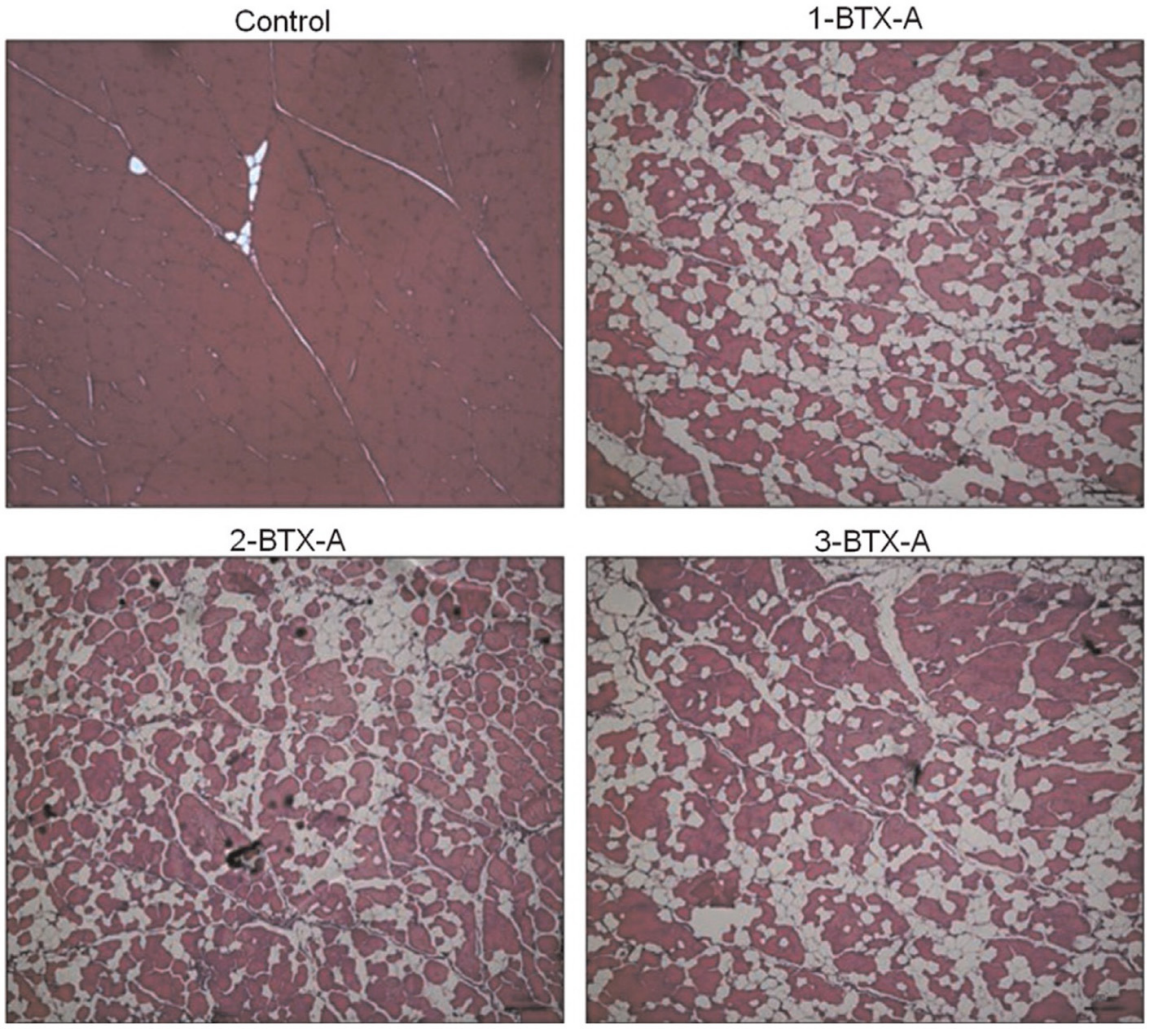
Exemplar histology slides of muscles injected with Botulinum toxin type-A (BoNT-A). Control tissue that was injected with a corresponding volume of saline (Control), muscle receiving one BTXA injection evaluated 6 months following injection (1-BTX-A), muscle receiving two BTXA injections separated by 3 months and evaluated 6 months post the second injection (2-BTX-A), and muscle injected three times with BTXA, separated by 3 months and evaluated 6 months following the third and last injection. The red staining shows the contractile material of the muscle. The white, thin lines (especially visible in the Control slide) are representative of the collagen matrix structure, and the white extended areas (especially visible in the BoNT-A injected muscles) represent intramuscular fat. The average percentage of contractile tissue in these muscles were 96.9 ± 2.0% (Control), 59.2 ± 6.0% (1-BTX-A), 62.5 ± 6.1% (2-BTX-A), and 59.9 ± 11.8% (3-BTX-A), respectively.

BoNT-A is a frequently used treatment modality aimed at reducing spasticity in children with cerebral palsy, with early basic science research purporting increased muscle growth (i.e., decreased muscle contracture) following intramuscular injection in a spastic mouse model (82, 83). The clinical effects of this treatment have been extensively described (84). However, relatively less attention has been paid to the short- and long-term effects of BoNT-A into the injected, target muscles and the potential side effects in noninjected, nontarget muscles. In a series of animal studies, we found that BoNT-A injections caused dramatic reductions in target muscle size and strength and were associated with greater intramuscular fat infiltration (85). Muscle strength was also reduced in adjacent, nontarget muscles, as was muscle size, strength, and substantial fibrosis in contralateral, noninjected muscles (52). Furthermore, a single, clinically relevant amount of BoNT-A injection has been shown to reduce muscle size in humans for up to a year (86) and produce a 50% decrease in strength and a decrease in contractile material with a corresponding amount of fat infiltration in rabbit knee extensor muscles after a 6 months recovery period (Figure 13) (57, 87). In addition, mRNA expression levels for the matrix molecules (responsible for fibrosis) collagens I and III, the anabolic growth factors insulin-like growth factor-1 (IGF-1) and transforming growth factor beta (TGFß), and muscle-specific atrophy marker MuRF1 were significantly elevated in the BoNT-A-injected animals compared to values in control group rabbits (57).

Human histological studies investigating the impact of BoNT-A on the CP skeletal muscle morphology are sparse at present, but two imaging studies deserve mention. In a study of 15 children with spastic diplegic CP receiving BoNT-A injections into the gastrocnemius, Williams et al. (88) used magnetic resonance (MR) imaging to measure calf muscle volumes 2 weeks before and 5 weeks after injection. In addition to a 5% decrease in gastrocnemius volume post-injection, they identified a concomitant 4% increase in soleus volume and suggested that this adjacent muscle hypertrophy may be compensatory. Another study by Schless et al. (89), applying ultrasound to the medial gastrocnemius, demonstrated decreased volume and increased connective tissue in children with CP compared to TDC, effects that were further exacerbated post BoNT-A injection.

In summary, BoNT-A injections in preclinical animal models have been shown to produce substantial and long-lasting muscle atrophy, muscle weakness, fat infiltration, fibrosis, inflammation, and degenerative molecular profiles that occur not only in target muscles but also in muscles close to the target injection site (within hours) and far removed from the injection site. More human, preferably histological, studies are needed to confirm these effects in the CP muscle.

### Surgical Management of Muscle Contracture

Surgical lengthening of muscle contractures is a long standing and widely accepted means to improve both muscle length and function in children with CP (90). Despite this history, there are few studies that specifically look at the effects of surgery on sarcomere length or function. On a more macroscopic scale, Shortland et al. (91) observed a post-operative reduction in both fascicle length and pennation angle in the medial gastrocnemius of children with spastic diplegic CP using ultrasound.

Also using ultrasound following postsurgical lengthening of semitendinosus (by tendon Z-plasty), Haberfehlner et al. (92) observed a 30% decrease in muscle belly length and an 80% increase in tendon length, implying a shortening of fascicles with a concomitant reduction in sarcomeres in series, despite improvements in knee joint kinematics. As discussed in previous sections, this would lead to a relative increase in sarcomere shortening during contraction and less ability to generate force. The authors suggested utilizing surgical strategies that maintained muscle belly length and cross-sectional area to help improve treatment outcomes.

Clearly, there is room for more studies investigating the role of surgery in CP muscle function, in particular with respect to its effects on the sarcomere.

### Other Treatments

The literature investigating muscle histopathology in CP after nonoperative treatments such as serial casting and physical therapy (PT) is currently sparse. Serial casting is a commonly used modality for the treatment of static muscle contracture in CP, based in early animal work that showed increased sarcomere addition following chronic stretch (93). In further animal work investigating soleus during stretch immobilization, sarcomere addition was not impaired in a satellite cell-depleted mouse model, despite demonstrating decreased muscle cross-sectional area (CSA) and increased extracellular matrix (ECM) connective tissue (94). The authors suggested that, although satellite cells likely play a role in the maintenance of the ECM and muscle volume, impaired sarcomerogenesis was not affected by the loss of these cells. Extrapolating these results to the use of serial casting in CP, it would seem that the increased length achieved is secondary to tendon length increases, rather than by sarcomere addition, and may be at the expense of muscle atrophy and ECM increase (95).

Ankle foot orthoses (AFOs) are commonly used in CP and other neuromuscular disorders, especially at night, to impose a chronic stretch to the gastrocnemius with the goal to improve muscle length and/or prevent static contracture. In the only clinical study investigating the impact of AFOs in CP, Hösl, and colleagues demonstrated decreases in fascicle length—which they suggested to be caused by reduced sarcomere addition—and little change in belly or tendon length (96).

The impact of PT, specifically passive stretching, on changes in CP muscle morphology at the fiber and myofibrillar levels is lacking at this point (97).

## Conclusions

Although research into the pathophysiology of the CP muscle is increasing, there are still many unanswered questions, particularly with respect to the development of fixed muscle contractures, reduced muscle growth, and weakness. The evidence to date, both from human and animal studies, would suggest that there are many factors at play, including satellite cell dysfunction, decreased myotube formation, increased fibrofatty tissue replacement, and overlong sarcomeres with reduced titin, possibly regulated by epigenetic instructions imprinted at the time of brain insult. Therapeutic measures, including BoNT-A and surgical lengthening, may serve to exacerbate muscle weakness in CP, with a dose-dependent effect. Additional studies investigating other treatments for spasticity reduction (e.g., selective dorsal rhizotomy), as well as properties of CP muscle with motor types other than spastic, are required to help complete the picture. Consideration of what is currently known about CP muscle pathophysiology by the treating clinician is important to help determine the most appropriate interventions for these children.

## Author Contributions

JH developed the initial review outline and wrote the clinically-based sections of the manuscript and also reviewed, edited, and formatted the final manuscript. WH revised the review outline and wrote the basic science-based sections of the manuscript and also reviewed, edited, and formatted the final manuscript. Both authors contributed to the article and approved the submitted version.

## Conflict of Interest

The authors declare that the research was conducted in the absence of any commercial or financial relationships that could be construed as a potential conflict of interest.
